# Ssp1 CaMKK: A Sensor of Actin Polarization That Controls Mitotic Commitment through Srk1 in *Schizosaccharomyces pombe*

**DOI:** 10.1371/journal.pone.0143037

**Published:** 2015-11-17

**Authors:** Alba Gómez-Hierro, Eva Lambea, David Giménez-Zaragoza, Sandra López-Avilés, Tula Yance-Chávez, Marta Montserrat, M. Jesús Pujol, Oriol Bachs, Rosa Aligue

**Affiliations:** 1 Departament de Biologia Cellular, Immunologia i Neurociències, Facultat de Medicina, Universitat de Barcelona, Institute of Biomedical Research August Pi i Sunyer (IDIBAPS), Barcelona, Catalunya, Spain; 2 The Biotechnology Centre of Oslo, Cell Cycle Regulation, Oslo, Norway; Hiroshima Universtiy, JAPAN

## Abstract

**Background:**

Calcium/calmodulin-dependent protein kinase kinase (CaMKK) is required for diverse cellular functions. Mammalian CaMKK activates CaMKs and also the evolutionarily-conserved AMP-activated protein kinase (AMPK). The fission yeast *Schizosaccharomyces pombe* CaMKK, Ssp1, is required for tolerance to limited glucose through the AMPK, Ssp2, and for the integration of cell growth and division through the SAD kinase Cdr2.

**Results:**

Here we report that Ssp1 controls the G2/M transition by regulating the activity of the CaMK Srk1. We show that inhibition of Cdc25 by Srk1 is regulated by Ssp1; and also that restoring growth polarity and actin localization of *ssp1*-deleted cells by removing the actin-monomer-binding protein, twinfilin, is sufficient to suppress the *ssp1* phenotype.

**Conclusions:**

These findings demonstrate that entry into mitosis is mediated by a network of proteins, including the Ssp1 and Srk1 kinases. Ssp1 connects the network of components that ensures proper polarity and cell size with the network of proteins that regulates Cdk1-cyclin B activity, in which Srk1 plays an inhibitory role.

## Introduction

Among the Ca^2+^/CaM-regulated enzymes found in eukaryotic cells, the multifunctional Ca^2+^/calmodulin-dependent protein kinases (CaMKs) occupy positions of influence because they communicate the Ca^2+^ signal via phosphorylation to a wide range of substrates [1,2]. As one of the many serine/threonine and tyrosine kinase families, the CaMK group is distinguished by its large number of constituent kinases [3–5]. Despite its nomenclature, however, only the classic CaMK subgroups such as the CaMKII family as well as the CaMKK and CaMKI/CaMKIV families, are genuinely catalytically Ca^2+^/CaM-dependent. Most of the kinases in the CaMK group lack the characteristic Ca^2+^/CaM-sensitive regulatory domain. They nonetheless belong to the CaMK group, because they share significant homology in the primary structure of their kinase domains [3–5]. In the genome of *S*. *pombe*, five genes code for proteins that have a high similarity with mammalian CaMK sequences: Cmk1, Cmk2 and Srk1 show sequence similarity to CaMKs and; while Ckk2 and Ssp1 shows sequence similarity with CaMKKs. Among these, only the activity of Cmk1 kinase has been proven to be Ca^2+^/CaM-dependent [6,7]. Cmk1 together with Ckk2 regulate cell growth in response to Ca^2^ [7]. Recently, the CaMKK Ckk2 has also been shown to be required for nitrogen-stress-induced AMP kinase activation (Ssp2) [8]. Cmk2 and Srk1 are related to the mammalian CaM-kinases and also to MAPKAP (MAP kinase-activated protein) kinases because they bind to and are activated by the MAPK (mitogen-activated protein kinase) p38/Sty1 [9–13]. Ssp1 also encodes a serine/threonine kinase with high similarity to the human CaM-kinase kinase (CaMKK) (42% identity) [14], described as the upstream activating kinase of CaMK [2]. Although there is no evidence of any Ca^2+^/CaM-dependent activity of Ssp1, it has recently been shown that Ssp1 has a conserved putative calmodulin binding domain (CBD) and a short stretch outside the kinase domain when compared to the amino acid sequences of human CaMKK1 and CaMKK2 [14]. In addition, Ssp1 shares a functional substrate with human CaMKKs, the AMP-activated protein kinase (Ssp2) [14–18]. Initially, Ssp1 kinase was reported to be required for growth polarity and actin localization at high temperature [19, 20]. Ssp1 mutants are unable to undergo the transition from monopolar to bipolar growth (new end take-off, NETO) and cells delay cell cycle progression into mitosis [19, 20]. NETO requires the completion of DNA replication and a critical cell size to be reached, indicating the existence of a signalling pathway that monitors these two requirements and regulates NETO during the cell cycle. Many of the genes involved in NETO have been identified and subsequently classified into four groups. Protein kinases constitute the largest group: Kin1 (Par-1/MARK-like), Pom1 (DYRK-like), Orb1 (PAK-like), Orb6 and Ssp1 [21, 22]. Ssp1 and Pom1 have recently been identified as part of a mechanism that controls cell growth and division, in which the SAD family kinase Cdr2 plays a key role [23]. Cdr2 promotes mitotic entry by inhibiting Wee1 kinase when the cell has reached the correct size. The activation of Cdr2 is achieved by the Ssp1 kinase through phosphorylation of a conserved threonine residue (Thr166) in the activation loop of the Cdr2 N-terminal kinase. Moreover, during cell growth, Pom1 also phosphorylates Cdr2 in the C-terminal domain, thus reducing Cdr2-T166 phosphorylation by Ssp1. Therefore, the activation of the mitotic inducer Cdr2 by Ssp1 is integrated with an inhibitory spatial gradient of Pom1 which ensures proper cell size control at mitosis.

Here, we studied whether the CaMKK Ssp1 regulates the fission yeast family of CaM-dependent kinases. We found that regulation of mitosis by Ssp1 is dependent on Srk1; we also found a link between both Ssp1 and the actin-binding protein Twf1, and the control of NETO. Srk1 and Twf1 can thus be added to the list of members of the network that controls the integration of cell growth and division.

## Material and Methods

### Fission yeast strains, media and techniques

The *S*. *pombe* strains used in this study are listed in Table 1.

**Table 1:** *Schizosaccharomyces pombe* strains.

Strain	Genotype	Source
RA2501	*h- leu1-32 ura4-D18*	Lab stock
MA6	*h- ssp1*::*ura4 leu1-32 ura4-D18*	Matsusaka et al.1995
RA1530	*h- srk1*::*kanMX6 leu1-32 ura4-D18*	Lab stock
RA1058	*h- srk1*::*kanMX6 ssp1*::*ura4 leu1-32*	This work
RA2663	*h- cmk1*::*kanMX6 leu1-32 ura4-D18*	Lab stock
RA0778	*h- cmk2*::*ura4 leu1-32 ura4-D18*	Lab stock
RA2502	*h- sty1*::*ura4 leu1-32 ura4-D18*	Lab stock
RA2726	*h- cmk1*::*kanMX6 ssp1*::*ura4 leu1-32*	This work
RA1057	*h- cmk2*::*ura4 ssp1*::*ura4 leu1-32*	This work
RA2236	*wee1*::*ura4 leu1-32 ura4-D18*	Lab stock
S1299	*h* ^*+*^ *cdc25-9A leu1-32*	H. Piwnica-Worms & P. San-Segundo
RA1973	*h- ssp1*::*ura4 cdc25-9A leu1-32*	This work
RA1949	*h- twf1*::*kanMX6 leu1-32 ura4-D18*	This work
RA2120	*h- ssp1*::*ura4 twf1*::*kanMX6 leu1-32 ura4-D18*	This work
RA1775	*h- for3*::*kanMX6 leu1-32 ura4-D18*	This work
RA1776	*h- ssp1*::*ura4 for3*::*kanMX6 leu1-32 ura4-D18*	This work
RA1809	*h- twf1*:*13myc*::*kanMX6 leu1-32 ura4-D18*	Lab stock
RA1970	*h- ssp1*::*ura4 aip3*::*kanMX6 leu1-32 ura4-D18*	This work
RA1964	*h- aip1*::*kanMX6 leu1-32 ura4-D18*	This work
RA1965	*h- ssp1*::*ura4 aip1*::*kanMX6 leu1-32 ura4-D18*	This work
RA0132	*h- wee1-50 leu1-32 ura4-D18*	Lab stock
RA118	*h- srk1*::*kanMX6 wee1-50 leu1-32 ura4-D18*	Lab stock
RA1943	*h- ssp1*::*ura4 wee1-50 leu1-32 ura4-D18*	Lab stock
RA3423	*h- ssp1*::*ura4 srk1*::*kanMX6 wee1-50 leu1-32 ura4-D18*	This work
RA3302	*h- srk1*::*kanMX6 cdr2*::*hphMX6 leu1-32 ura4-D18*	This work
RA3303	*h- ssp1*::*ura4 srk1*::*kanMX6 leu1-32 ura4-D18*	This work
RA3304	*h- ssp1*::*ura4 srk1*::*kanMX6 cdr2*::*hphMX6 leu1-32 ura4-D18*	This work

General yeast techniques and manipulations were carried out as previously described [24]. Cells were grown either in YE (yeast extract) medium or Edinburgh minimal medium (EMM) with appropriate supplements. All strains were cultured at 30°C except during temperature-sensitive assays, during which strains were grown either at the permissive temperature of 25°C or at the restrictive temperature of 36°C as indicated. *S*. *pombe* transformations were carried out using either a lithium acetate method [25] or electroporation [26]. Gene deletion and epitope tagging were carried out as described elsewhere [27]. DNA was prepared from bacteria and isolated from agarose gels using Qiagen kits.

### Immunochemical analysis and microscopy

Cells were grown from 6 h to overnight at 36°C, fixed with methanol at -20°C, mounted with Mowiol (Calbiochem), and cell imaging was performed under a Leica SP5 Confocal Microscope. For actin staining, cells were fixed with formaldehyde 60%, washed twice in PM Buffer (35 mM K-Phos pH 6.8, 0.5 mM MgSO_4_), permeabilized with 1% Triton X-100, washed twice in PM Buffer, and stained with phalloidin conjugated-Alexa Fluor 488 (Invitrogen, Molecular probes) for 40 min in the dark. Cells were mounted and cell imaging was performed under a Leica SP5 Confocal Microscope. Image analysis and measurements were carried out using Image J.

### Immunoprecipitation and Western blotting analysis

Aliquots of 1 x 10^8^ cells were lysed in buffer (150 mM NaCl, 50 mM Tris-HCl [pH 8.0], 5 mM EDTA, 0.1% Triton X-100, 10% glycerol, 50 mM NaF, 1 mM PMSF, 1 mM NaVO_4,_ 5 μg/ml aprotinin, 5 μg/ml leupeptin). Protein immunoprecipitation was performed from cell extracts with either protein A or protein G Sepharose beads, and immunoprecipitates were washed four times in lysis buffer prior to analysis. Proteins were resolved by SDS-polyacrylamide gel electrophoresis (SDS PAGE) and analyzed by Western blotting. The following primary antibodies were used: polyclonal anti-Cdc25 (1/1000), monoclonal anti-HA (12CA5, Roche, Indianapolis, IN; 1/1000); polyclonal anti-PSTAIR (Upstate Biotechnology, Lake Placid, NY; 1/1000), and monoclonal anti-myc (9E10; 1/1000). Horseradish peroxidase conjugated anti-mouse or anti-rabbit antibodies (Bio-Rad, Richmond, CA) were used as secondary antibodies. Membranes were developed by enhanced chemiluminescence (ECL kit, Amersham-Pharmacia, Piscataway, NJ).

## Results

### Deletion of Srk1 kinase suppresses the mitotic delay of *ssp1Δ*

**Fig 1:**
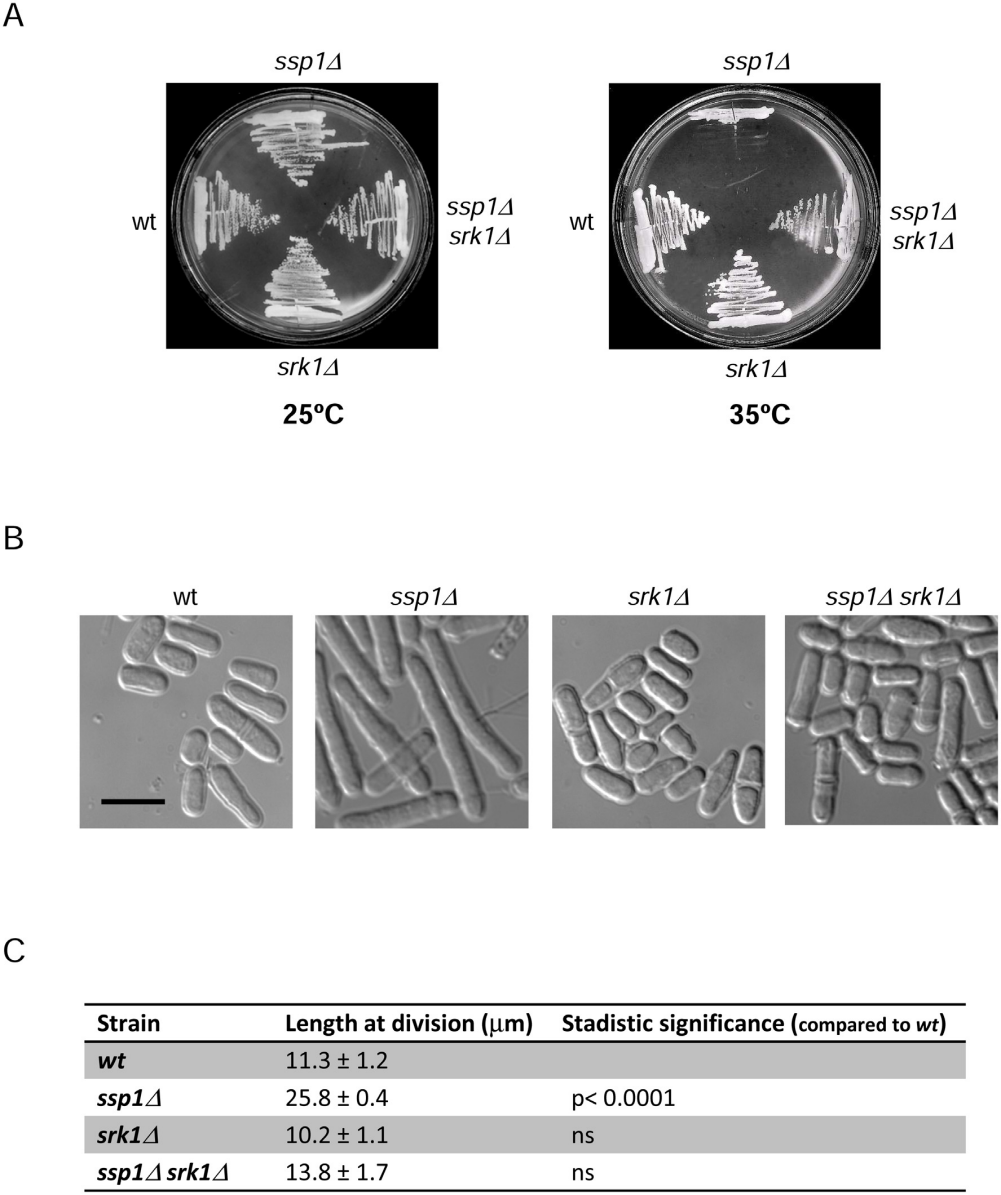
Srk1 deletion rescues cell cycle arrest due to the absence of Ssp1. A. Wild-type (wt), *ssp1Δ*, *srk1Δ* and *ssp1Δ srk1Δ* cells were grown on YES plates for 3 days at 25°C and 35°C. B. Wild-type (wt), *ssp1Δ*, *srk1Δ* and *ssp1Δ srk1Δ* cells were grown at 25°C in YES liquid medium to mid-log phase before being transferred to 35°C for 9 hours and visualized microscopically. Scale bar, 10 μm. C. Length of dividing septated cells of the indicated strains (mean ± SD; n >50 for each value). Stadistic significance compared to wild type performed by t-test analysis of 3 values.

To assess the interaction between Ssp1 and previously identified CaMK homologues in fission yeast, we created double mutants between *ssp1Δ* and the kinases *cmk2Δ*, *srk1Δ* and *cmk1Δ*. Their effects were analyzed at 35°C, at which the cell division of *ssp1Δ* is arrested, leading to an elongated phenotype. Only *srk1Δ* rescued the cell division arrest of *ssp1Δ* cells (Fig 1A and S1 Fig). The cell elongation phenotype of *ssp1Δ* cells was also rescued by *srk1Δ* (Fig 1B).

### Srk1 operates downstream of Ssp1 activity

**Fig 2:**
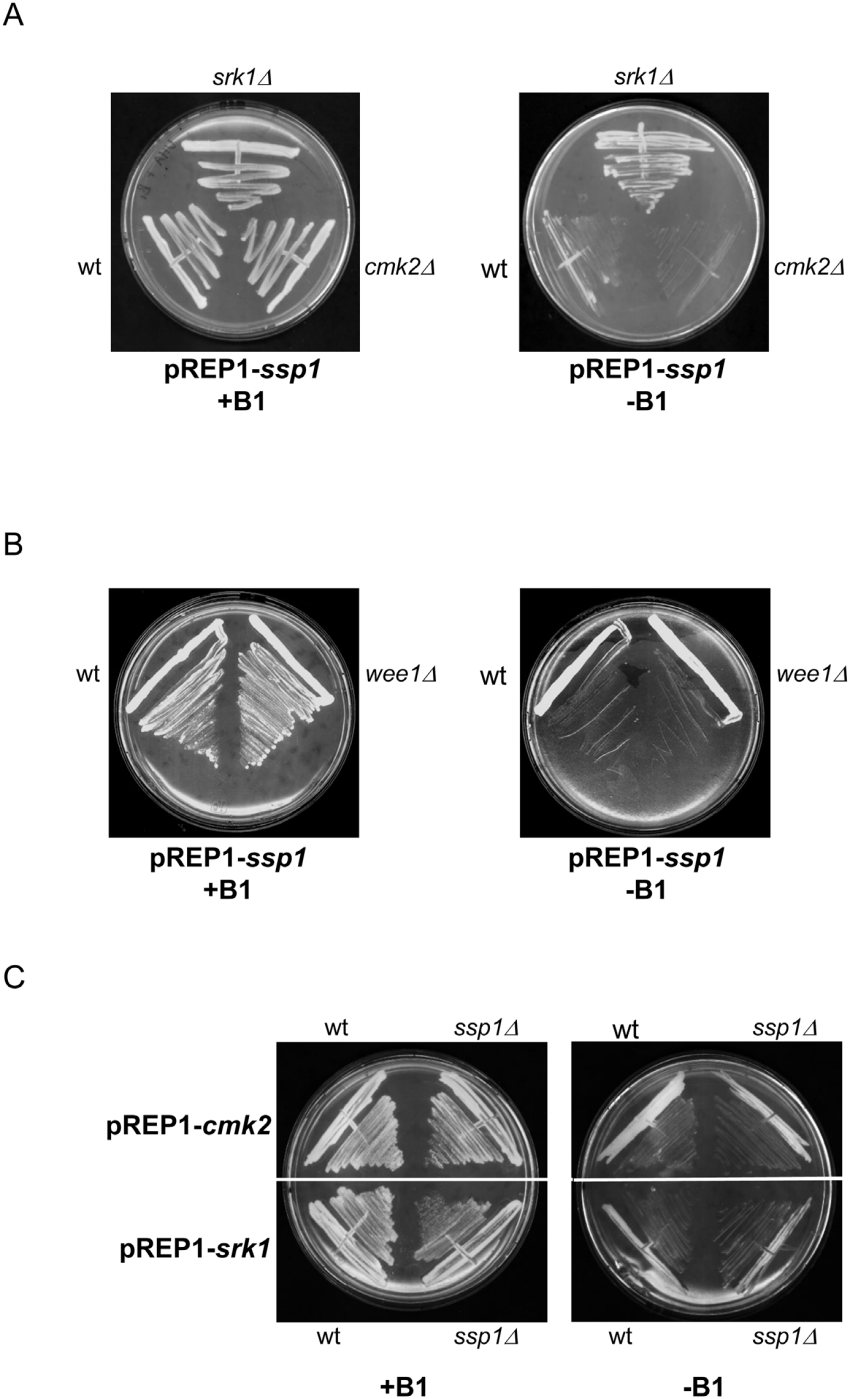
Ssp1 acts upstream of Srk1. A. Wild-type, *srk1Δ* and *cmk2Δ* cells transformed with pREP1-*ssp1* were grown in liquid culture cells, either in the absence of thiamine (-B1) or presence of thiamine (+B1) for 3 days. B. Wild-type and *wee1Δ* cells transformed with pREP1-*ssp1* were grown in liquid culture cells, either in the absence of thiamine (-B1) or presence of thiamine (+B1) for 3 days. C. Wild-type and *ssp1Δ* cells transformed with pREP1-*cmk2* and pREP1-*srk1* were grown in liquid culture cells, either in the absence of thiamine (-B1) or presence of thiamine (+B1) for 3 days.

To evaluate whether Srk1 interferes with Ssp1 activity, the slow cell growth exhibited by overexpression of Ssp1 was analyzed in *srk1Δ* and *cmk2Δ* cells. Only *srk1Δ* cells suppressed the slow growth related with Ssp1 overexpression (Fig 2A), indicating that Srk1 is necessary for Ssp1 activity. In order to rule out the possibility that the deletion of any negative regulator of mitosis could suppress the Ssp1 slow growth phenotype, Ssp1 was also overexpressed in *wee1Δ* cells. Deletion of *wee1* did not suppress the slow growth related with Ssp1 overexpression (Fig 2B).

Srk1 was overexpressed in *ssp1Δ* cells and the cell cycle arrest caused by the overexpression of Srk1 was not abolished, indicating that Ssp1 is upstream of Srk1 (Fig 2C). The same was done with Cmk2, and as shown in Fig 2C, deletion of *ssp1* did not abolish the cell cycle arrest caused by Cmk2, showing that Cmk2 and Ssp1 are independent.

### Deletion of Ssp1 increases the abundance of Cdc25 in a Srk1-dependent manner

**Fig 3:**
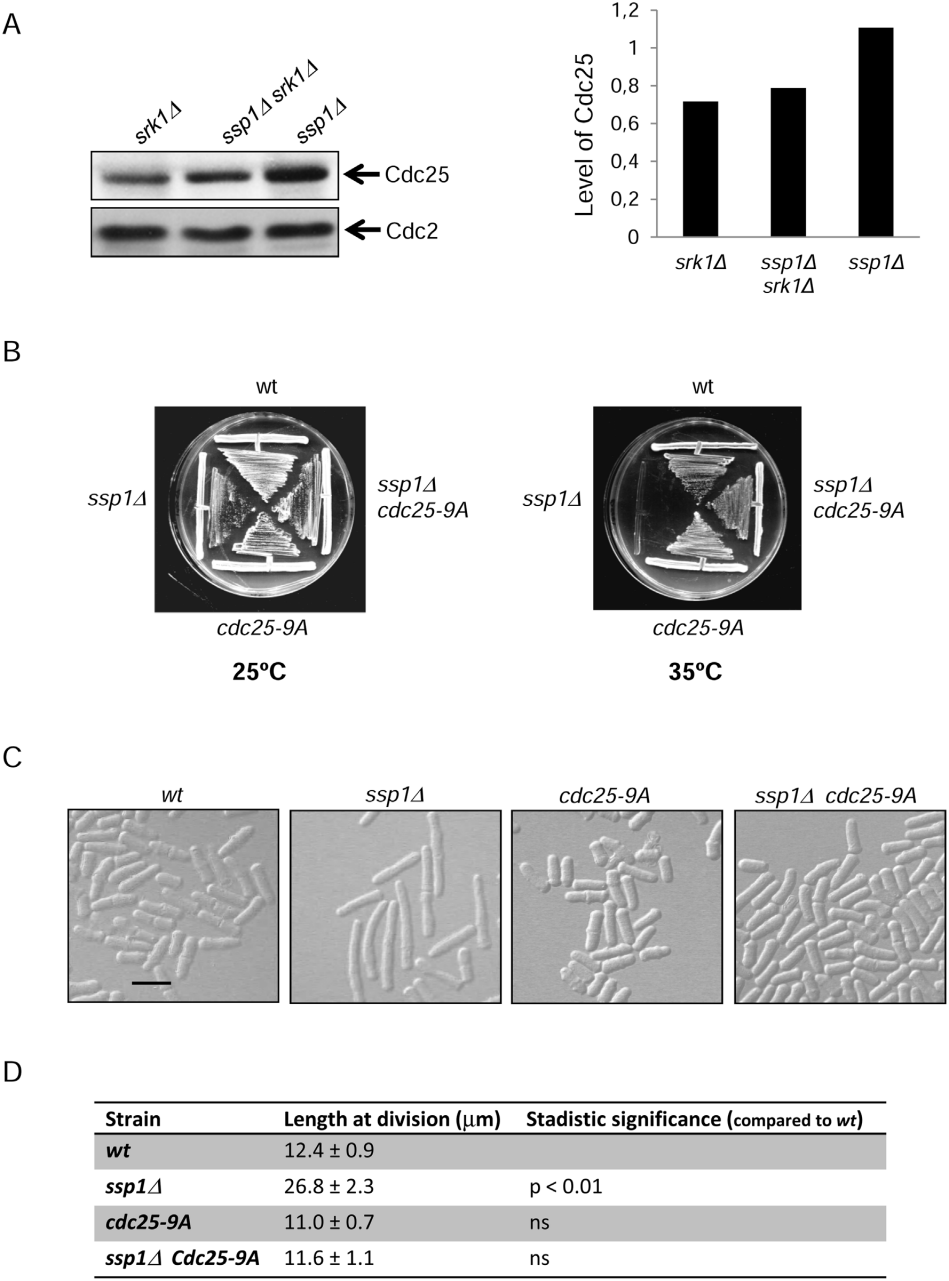
Srk1 is activated in *ssp1Δ* cells, causing accumulation and phosphorylation of Cdc25. A. Cell extracts were prepared from *ssp1Δ*, *srk1Δ* and *ssp1Δ srk1Δ* cells and analyzed by Western blot to monitor the levels of Cdc25 with anti-Cdc25 (top) or Cdc2 with anti-PSTAIR antibodies as a loading control (bottom). The graph represents the quantification of the Cdc25 protein level regarding the Cdc2 load control. B. Wild-type (wt), *ssp1Δ*, *cdc25-9A* and *ssp1Δ cdc25-9A* cells were grown in YES media for 3 days at 25°C and 35°C. C. Wild-type (wt), *ssp1Δ*, *cdc25-9A* and *ssp1Δ cdc25-9A* cells were grown at 25°C in YES liquid medium to mid-log phase before being transferred to 35°C for 12 hours and visualized microscopically. Scale bar, 10 μm. D. Length of dividing septated cells of the indicated strains (mean ± SD; n >50 for each value). Stadistic significance compared to wild type performed by t-test analysis of 3 values.

Srk1 inhibits the G2/M transition by phosphorylation of Cdc25, which provokes Cdc25 stabilization through binding to the 14.3.3 protein Rad24 [10]. The fact that the *srk1* mutant suppressed the cell cycle delay of *ssp1Δ* suggests that Srk1 was activated and consequently, inhibited the cell cycle progression in *ssp1Δ* cells. To test the activation of Srk1 we analyzed the levels of Cdc25 protein in *ssp1Δ* and *ssp1Δ srk1Δ* cells compared to the levels in *srk1Δ* cells as negative control. Cdc25 protein levels were stable, being increased in *ssp1Δ* cells and decreased in the double mutant *ssp1Δ srk1Δ* as in *srk1Δ* cells (Fig 3A).

To further investigate whether Srk1 delays the *ssp1Δ* cell cycle by inhibiting Cdc25, we assessed the cell cycle progression of *ssp1Δ cdc25-9A* double mutant cells, in which the endogenous *cdc25* gene has nine Srk1-phosphorylation sites mutated to alanine. We observed that mutation of Srk1-phosphorylation sites of Cdc25 abolished the cell cycle arrest of *ssp1Δ* (Fig 3B). The cell elongation phenotype of *ssp1Δ* cells was also rescued by *cdc25-9A* (Fig 3C and 3D). We conclude that Srk1 inhibitory activity is required for cell division arrest in *ssp1Δ* cells.

### Ssp1 is necessary to maintain cell viability in the absence of Wee1 and Srk1

**Fig 4:**
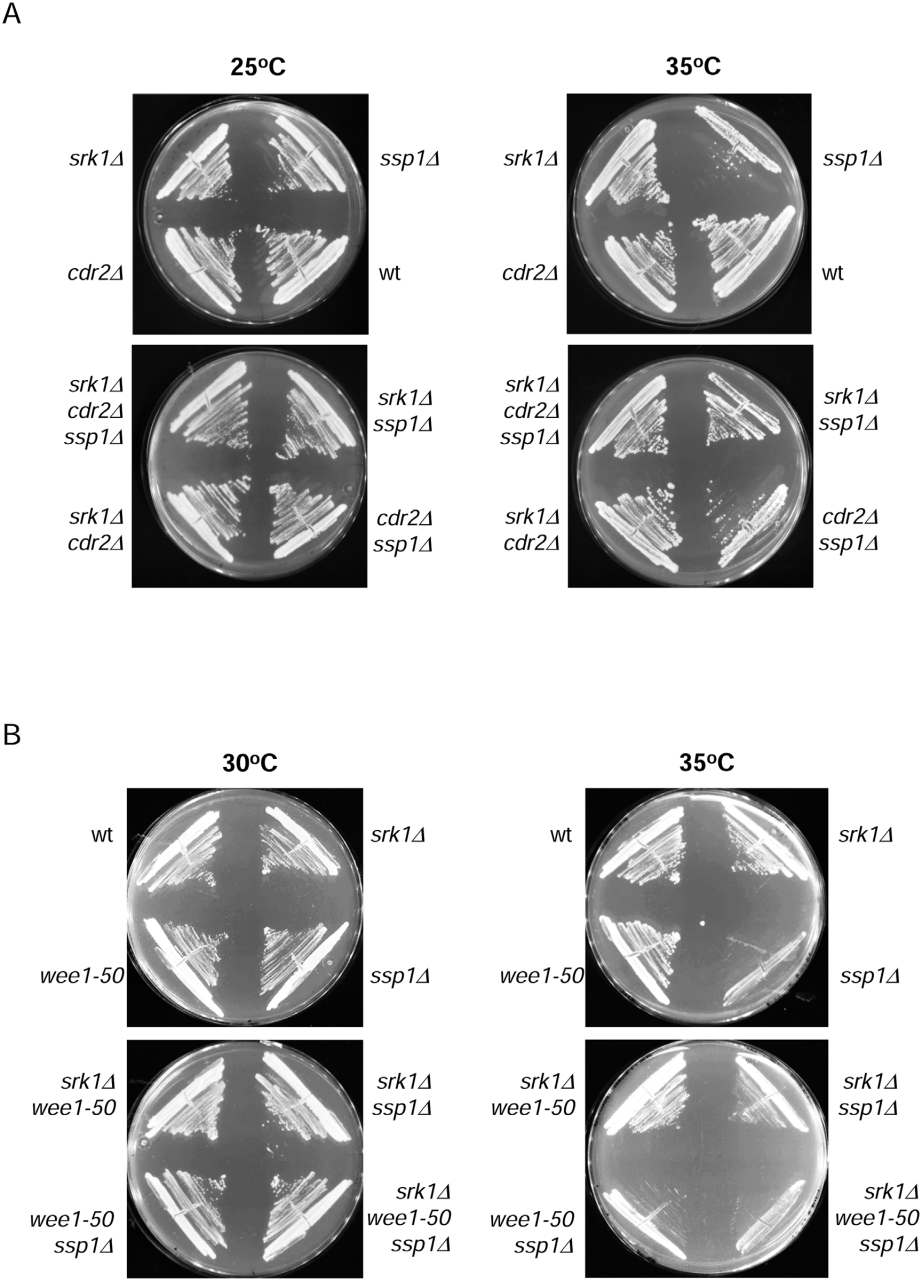
The rescue of the slow growth of *ssp1* deletion by *srk1Δ* is abolished by the absence of *wee1*. A. Wild-type (wt), *ssp1Δ*, *srk1Δ*, *cdr2Δ* and the double mutants *ssp1Δ srk1Δ*, *ssp1Δ cdr2Δ*, *srk1Δ cdr2Δ* and the triple mutant *ssp1Δ srk1Δ cdr2Δ* cells were grown in YES plates for 3 days at 25°C and 35°C. B. Wild-type (wt), *ssp1Δ*, *srk1Δ*, *wee1-50* and the double and triple mutants *ssp1Δ wee1-50*, *srk1Δ wee1-50*, *ssp1Δ srk1Δ*, and *ssp1Δ srk1Δ wee1-50* cells were grown in YES plates for 3 days at 30°C and 35°C.

It was recently reported that Ssp1 activates Cdr2 which in turns inactivates Wee1 kinase when cells reach mitotic size [23]. We have analyzed the cell growth of the double mutant *ssp1Δ cdr2Δ* at 35°C; as expected, cell growth was arrested as in *ssp1Δ* cells (Fig 4A). Moreover when *srk1* was deleted from the *ssp1Δ cdr2Δ* cells, cell growth was restored (Fig 4A).

We next analyzed whether the absence of *wee1* kinase rescued cell growth arrest in *ssp1Δ* cells. As Fig 4B shows, cell growth is not rescued by the loss of *wee1* (wee1-50 mutant). We also examined the double mutant *wee1-150 srk1Δ*; it was interesting to observe that cells growth better than *wee1-50 or srk1Δ* single mutants (Fig 4B). When *ssp1* was deleted in the double mutant *wee1-150 srk1Δ* cell growth was arrested (Fig 4B).

### Bipolar growth defects of *ssp1Δ* cells are independent of Srk1

**Fig 5:**
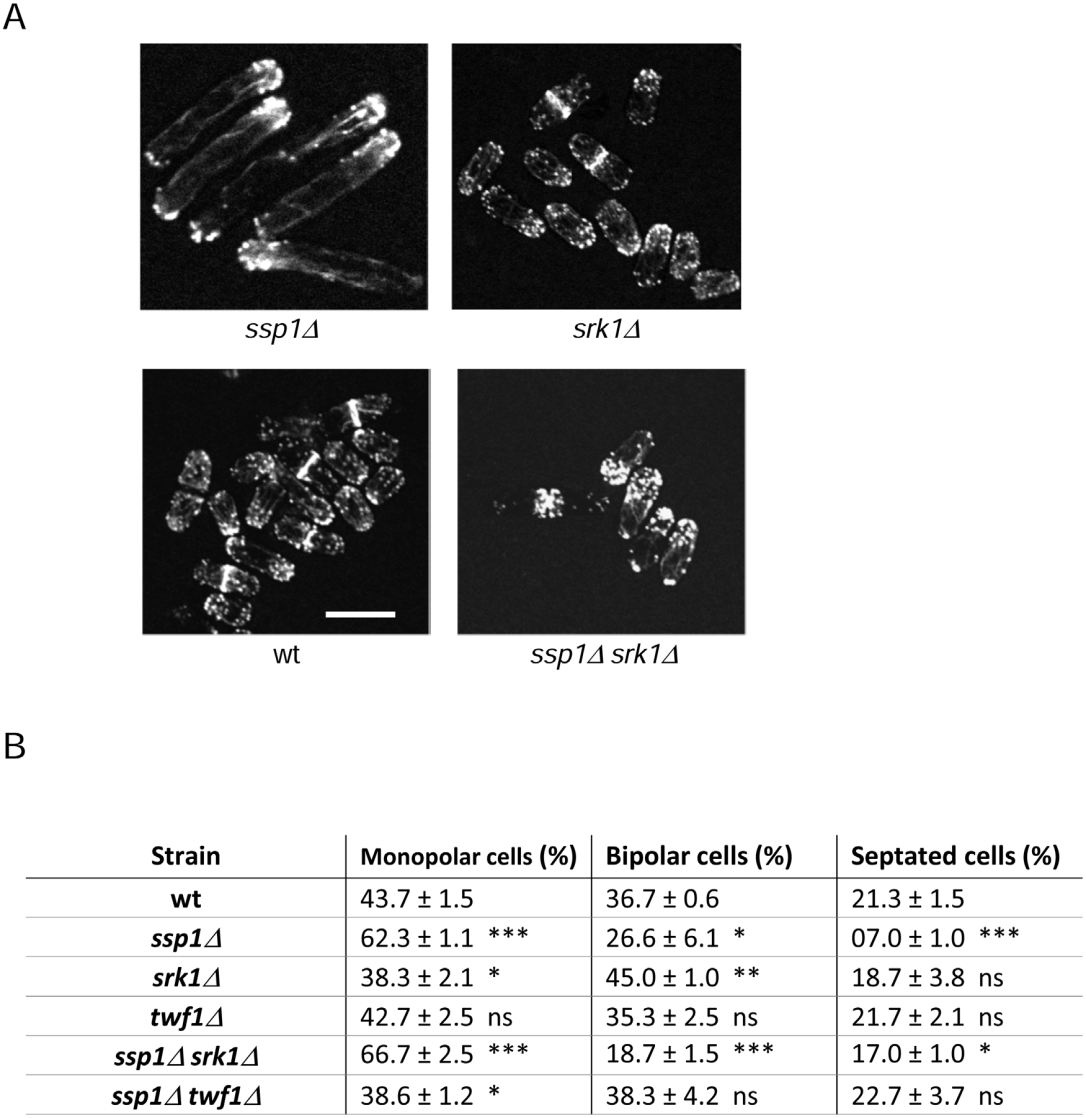
Monopolar actin distribution in double *srk1 ssp1*-deleted cells. A. Localization of actin in wild-type, *ssp1Δ*, *srk1Δ* and *ssp1Δ srk1Δ* cells grown at 35°C for 12 hours and visualized microscopically, single focal planes. Scale bar, 10 μm. B. Frequency of growth polarity after 9 hours at 35°C. Next to the frequency is shown the stadistic significance compared to wild-type by T-test of 3 values of each strain. The p value of the symbols is p > 0.05 (ns), p < 0.05 (*), p < 0.01 (**), p < 0.001 (***) and p < 0.0001 (****).

Ssp1 is required for the efficient initiation of a second site of polarized growth at the NETO [19, 20]. We analyzed whether Srk1 is involved in this Ssp1 function by examining the actin localization in the double mutant *ssp1Δ srk1Δ* cells. As shown in Fig 5, actin patches were accumulated in one cell tip in *ssp1Δ srk1Δ* cells as in the single mutant *ssp1Δ* (Fig 5A and 5B). Thus, the monopolar growth of *ssp1Δ* cells is not controlled by Srk1 activity.

### Deletion of twinfilin (*twf1*) rescues *ssp1Δ* bipolar growth defects

**Fig 6:**
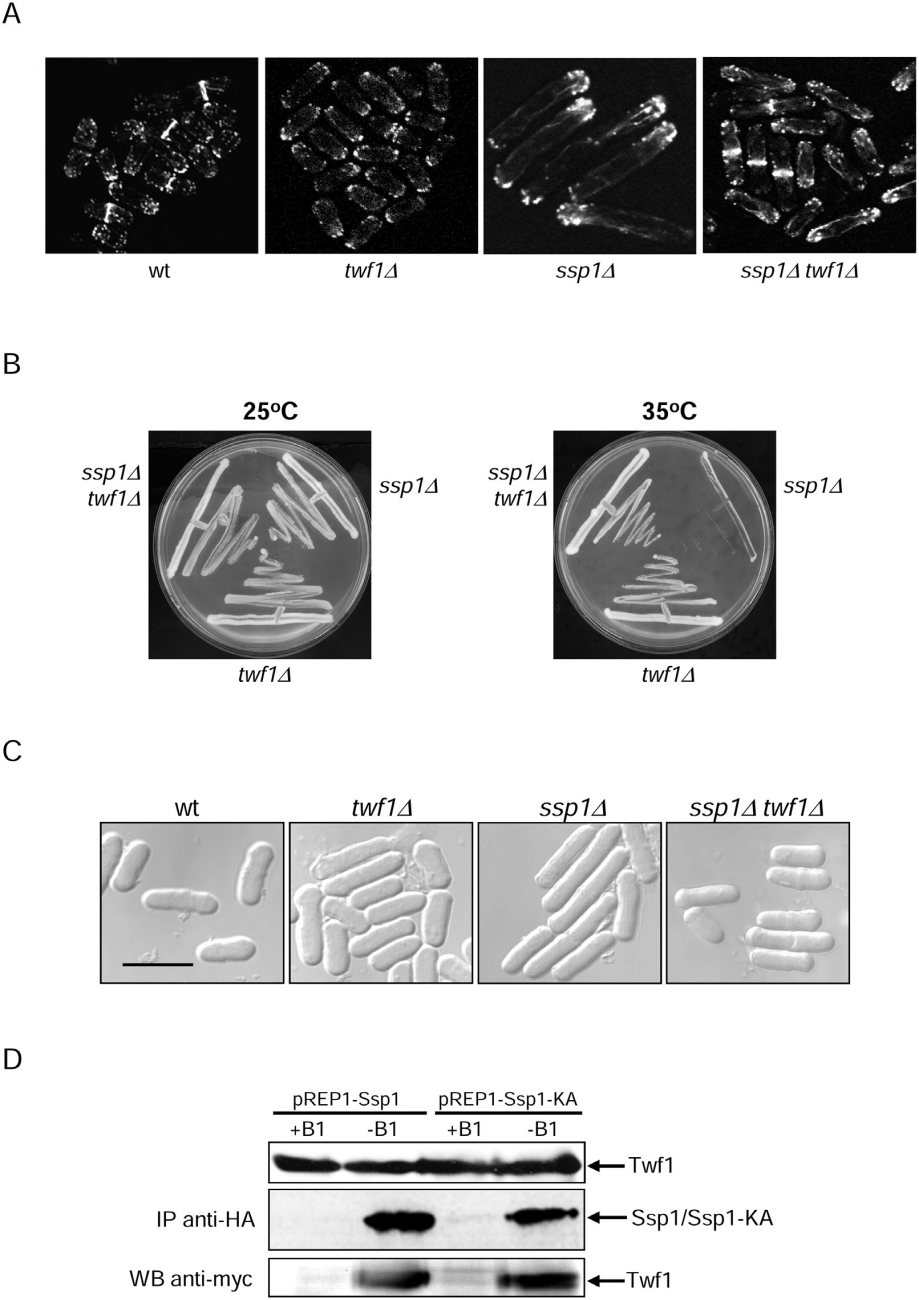
Absence of twinfilin restores bipolar actin localization and cell division of *ssp1-*deleted cells. A. Localization of actin in wild-type, *ssp1Δ*, *twf1Δ* and *ssp1Δ twf1Δ* cells grown at 35°C for 12 hours and visualized microscopically, single focal planes. Scale bar, 10 μm. B. Wild-type (wt), *ssp1Δ*, *twf1Δ* and *ssp1Δ twf1Δ* cells were grown on YES plates for 3 days at 25°C and 35°C. C. Wild-type (wt), *ssp1Δ*, *srk1Δ* and *ssp1Δ srk1Δ* cells were grown at 25°C in YES liquid medium to mid-log phase before being transferred to 35°C for 9 hours and visualized microscopically. Scale bar, 10 μm. D. Ssp1 interacts with Twf1. Twf1-9myc cells were transformed with pREP1-*ssp1* and pREP1-*ssp1-KA* and grown in the presence (+B1) or absence (-B1) of thiamine. Ssp1-HA was immunoprecipitated from cells extracts and analyzed by Western blot for the presence of Ssp1 and Twf1 with anti-HA and anti-myc antibodies, respectively.

*ssp1Δ* cells show the monopolar localization of actin patches and therefore monopolar growth. It is also known that activation of actin mobilization is sufficient for reestablishing bipolar growth in *ssp1Δ* cells [19]. Twinfilin (Twf1) is an actin-monomer-binding protein that inhibits nucleotide exchange on actin monomers and prevents assembly of the monomers into filaments [28]. Therefore, we investigated whether the Twf1 protein is involved in Ssp1-actin dynamic regulation. We analyzed the actin localization of *twf1Δ* and the double mutant *ssp1Δ twf1Δ* cells and found that deletion of *twf1* suppressed the monopolar localization of the actin patches in *ssp1Δ* cells, thus activating NETO (Figs 6A and 5B).

We next analyzed whether *twf1Δ* rescued the *ssp1Δ* growth defect at 35°C and indeed, deletion of *twf1* suppressed *ssp1Δ* lethality at 35°C (Fig 6B).

Analysis of the cell length also indicated that *twf1Δ* suppressed the cell cycle delay of *ssp1Δ* cells manifested as an elongated phenotype (Fig 6C).

To study whether Ssp1 regulates Twf1 directly, the interaction between Ssp1 and Twf1 was analyzed. Cells co-expressing Ssp1 or Ssp1-KA (catalytically inactive Ssp1) and Twf1 tagged with different epitopes (Ssp1/Ssp1-KA-HA and Twf1-myc respectively) were pulled down from exponentially growing yeast cell extracts. Ssp1/Ssp1-KA was immunoprecipitated with anti-HA-beads and the presence of Srk1-myc was examined by Western blot. As Fig 6D shows, Twf1 was pulled down together with Ssp1 and also with the catalytically inactive Ssp1-KA.

## Discussion

### Srk1 activity in G2/M transition is dependent on Ssp1

The activation of Cdk1-cyclin B and the subsequent triggering of mitosis is controlled by the phosphorylation status of the Cdk1 catalytic subunit. Cdk1 phosphorylated by Wee1 blocks mitosis activation until Cdc25 phosphatases remove the phosphate to drive division. Moreover, entry into mitosis is mediated by a network of proteins that regulate the activation of the Cdk1 complex. Within this network, several components act to swing the balance to a mitotic commitment by ensuring the complete activation of Cdk1. Here, we report that the CaMKK Ssp1 is one of the players that ensures activation of Cdk1 by negatively controlling Srk1 and thereby allowing Cdc25 activation. Deletion of *ssp1* arrests the cell cycle in the G2/M transition. This cell cycle arrest appears to stem from Srk1 activation. This is supported by our results showing that deletion of *srk1* rescues cell cycle progression of the arrested *ssp1Δ* cells.

The activation of Srk1 provokes Cdc25 phosphorylation; Cdc25 activity is consequently inhibited and the Cdc25 protein stabilized [10, 11]. The stabilization of Cdc25 ensures its rapid incorporation and the activation of Cdk1 after the arrest [10]. We showed that the Cdc25 protein is stabilized, and thus protein levels increased, in *ssp1*-depleted cells, and decreased when *srk1* was removed from these cells. This indicates that the maintenance of Cdc25 protein is dependent on Srk1 kinase. Further evidence that cell cycle arrest in *ssp1*-deleted cells is due to Cdc25 inhibition dependent on Srk1 activity was provided by the finding that mutation of Srk1-dependent phosphorylation sites in Cdc25 was sufficient to rescue the cell cycle arrest of *ssp1Δ* cells.

Furthermore, we also analyzed the overexpression of Ssp1 in the *srk1* mutant. Ssp1 overexpression caused slow growth and an abnormal phenotype, cells showed a pear or round shape, due to the actin misallocation by Ssp1 [19]. The slow growth and abnormal phenotype resulting from Ssp1 overexpression was rescued by deleting *srk1*. This observation does not correlate with the role of Ssp1 in the cell cycle by regulating negatively Srk1. Accordingly, overexpression of Ssp1 in *srk1* deleted cells should be the same as overexpression in wild type cells; but that was not the case. However this observation indicates that Srk1 is necessary for the Ssp1 morphological function regarding the cytoskeketon.

Mitotic commitment is integrated with cell size control. The key to this integration is the SAD family kinase Cdr2, which organizes cortical nodes in the center of the cell and promotes mitotic entry through the inhibition of Wee1 [29, 30]. It was recently reported that Ssp1 activates Cdr2 through the phosphorylation of a conserved threonine residue (T166) in the activation loop. The level of Cdr2-threonine 166 phosphorylation increased along the cell cycle and was reduced in a Pom1-dependent manner, before the cells reached mitotic size [23, 31]. Interestingly, while the double *cdr2-T166A ssp1Δ* mutant cells showed the same phenotype as *ssp1Δ* cells, the double mutant *cdr2Δ ssp1Δ* showed an enhanced phenotype. Cells were longer than in the *cdr2-T166A ssp1Δ* mutant [23], indicating that the Ssp1-Cdr2 axis must exert additional cell cycle control. This observation correlates with our results which indicates that Srk1 activity is connected with the Ssp1-Cdr2 axis and cell size control, as deletion of *srk1* rescues cell growth of the double mutant *cdr2Δ ssp1Δ*. Therefore, Ssp1 kinase causes the onset of mitosis via activation of Cdr2 and Cdc25, by controlling Srk1 activity. Moreover, if Ssp1 inhibits Wee1 through Cdr2, we may expect that *wee1* deletion would rescue *ssp1Δ* cell cycle arrest; but that was not the case. Even when cell cycle was accelerated due to the loss of both the *wee1* and *srk1* genes, deletion of *ssp1* blocked cell cycle progression. This observation points the role of Wee1 in cell morphology together with Ssp1.

### Ssp1 operates as a sensor of actin flowthrough

Ssp1 has also been identified as being necessary for the initiation of growth at the new cell end (NETO) following division and stress-induced reorganization of the actin cytoskeleton in fission yeast [19, 20]. The *ssp1*-deleted cells exhibited a monopolar actin distribution. Although our data revealed that deletion of *srk1* rescues the cell cycle arrest of *ssp1Δ* cells, it did not reestablish bipolar growth; indicating that Srk1 is not involved in Ssp1-actin allocation during cell division. It was reported that NETO could be induced in *ssp1Δ* cells by exposure to KCl or latrunculin A pulse treatment; both of which induce a transient redistribution of actin monomers [20]. The experiments with this, we identified twinfilin as the actin-monomer-binding protein involved in the bipolar-growth function of Ssp1. Surprisingly, restoring the actin monomer stream was sufficient to rescue cell cycle arrest of *ssp1Δ*. We tested additional actin regulatory proteins such as formin (For3, Aip1 and Aip3) [32–34] to examine whether the absence of any actin-nucleator or actin-binding protein would rescue cell cycle arrest of *ssp1Δ*, but found that the deletion of For3, Aip1 or Aip3 had no effect (S2 Fig).

Twinfilin is present in eukaryotes from yeasts to mammals. Despite its sequence homology with ADF/cofilin, twinfilin binds to monomeric actin and does not promote actin-filament depolimerization [35]. Twinfilin is an abundant protein that localizes in cortical actin patches in wild-type yeast cells and this localization is dependent on a direct interaction with capping proteins [36]. Twinfilin also interacts with phosphatidyliniositol 4,5-biphosphate (IP[4,5]P2) and its actin monomer-sequestering activity is inhibited by IP(4,5)P2 [36]. However, the mechanistic role of twinfilin in actin disassembly is unclear [37]. Mutations of twinfilin, in budding yeast and *Drosophila*, result in an enlargement of cortical actin patches and defects in actin-dependent developmental processes, respectively [38]. Furthermore, deletion of twinfilin from budding yeast is synthetically lethal with certain cofilin and profilin mutations; this indicates its role in the regulation of actin dynamics *in vivo* [38].

Interestingly, in mammalian cells, twinfilin has been identified as a target of the microRNA-30c (miR-30c) [39, 40] and RunX2 transcription factor [41]. In breast cancer cells, twinfilin promotes epitheliat to mesenchymal transition and together with miR-30c regulates invasion and chemoresistance [39, 40]. In addition, twinfilin regulates the expression of interleukin 11 (IL-11) at both the mRNA and protein levels; although the detailed mechanisms has yet to be elucidated [39]. It has been proposed, that twinfilin regulates IL-11 through sequestering the actin monomers pool; as a consequence, a reduced actin monomer pool could release actin-bound specific transcription factors, thereby activating its nuclear translocation [39].

Moreover, twinfilin was found to be upregulated in genome-wide mRNA expression changes in prostate cancer cells in response to Runx2 [41]. Runx2 is an osteoblast master transcription factor that is aberrantly expressed in prostate cancer cells and promotes their metastatic phenotype. The major functions reported for the genes up-regulated by Runx2 belonged to cancer progression. These genes encode transcriptional regulators, signaling molecules, peptidases involved in tumor metastasis and actin cytoskeleton dynamics, where twinfilin was included [41].

In fission yeast, nothing is known about the expression or dynamics of Twf1 in cell cycle progression. It is possible that Twf1 regulates the cellular localization of specific transcription factors associated with the actin cytoskeleton. Therefore, Twf1, by sequestering actin monomers, may help to release the transcription factors and allow their nuclear translocation; in a way similar to that proposed in mammalian cells. However, neither do we know the mechanism by which Ssp1 influences Twf1. Our results show that Twf1 and Ssp1 coprecipitate, this association could be located at the membrane. Since, Ssp1 localizes at the plasma membrane under stress conditions [20, 42] and Twf1 binds IP(4,5)P which inhibits its monomer-sequestering activity [36]. Further studies will be necessary to reveal the specific mechanism of Twf1 regulation and its role in cell cycle control.

Overall, our data suggest that Ssp1 is part of a complex of proteins that control cell cycle progression, such as Srk1 and Cdr2, as well as proteins that regulate actin polarization, such as Twf1. It is thus proposed that Ssp1 is a sensor of actin flow to regulate cell cycle progression.

## Supporting Information

S1 Fig Interaction between Ssp1 and the CaMKs, Cmk2 and Cmk1 kinases. Wild type (wt), *cmk1Δ*. *cmk2Δ*, *ssp1Δ*, *cmk1Δ ssp1Δ*, *cmk2Δ ssp1Δ* and *sty1Δ* cells were grown in YES medium and spotted on YES plates and incubated for 3 days at 25°C and 35°C.
(TIF)

S2 Fig Interaction of the Ssp1 with actin regulatory proteins. (A) *ssp1Δ*, *for3Δ* and *ssp1Δ for3Δ* cells were grown on YES plates for 3 days at 25°C and 35°C. (B) *ssp1Δ*, *aip1Δ* and *ssp1Δ aip1Δ* cells were grown on YES plates for 3 days at 25°C and 35°C. (C) *ssp1Δ*, *aip3Δ* and *ssp1Δ aip3Δ* cells were grown on YES plates for 3 days at 25°C and 35°C.
(TIF)
