# Supplementary figures and images for: Ssp1 CaMKK: A Sensor of Actin Polarization That Controls Mitotic Commitment through Srk1 in Schizosaccharomyces pombe
Source: PLoS One. 2015 Nov 17;10(11):e0143037. doi: 10.1371/journal.pone.0143037 (PMC4648557; doi:10.1371/journal.pone.0143037)

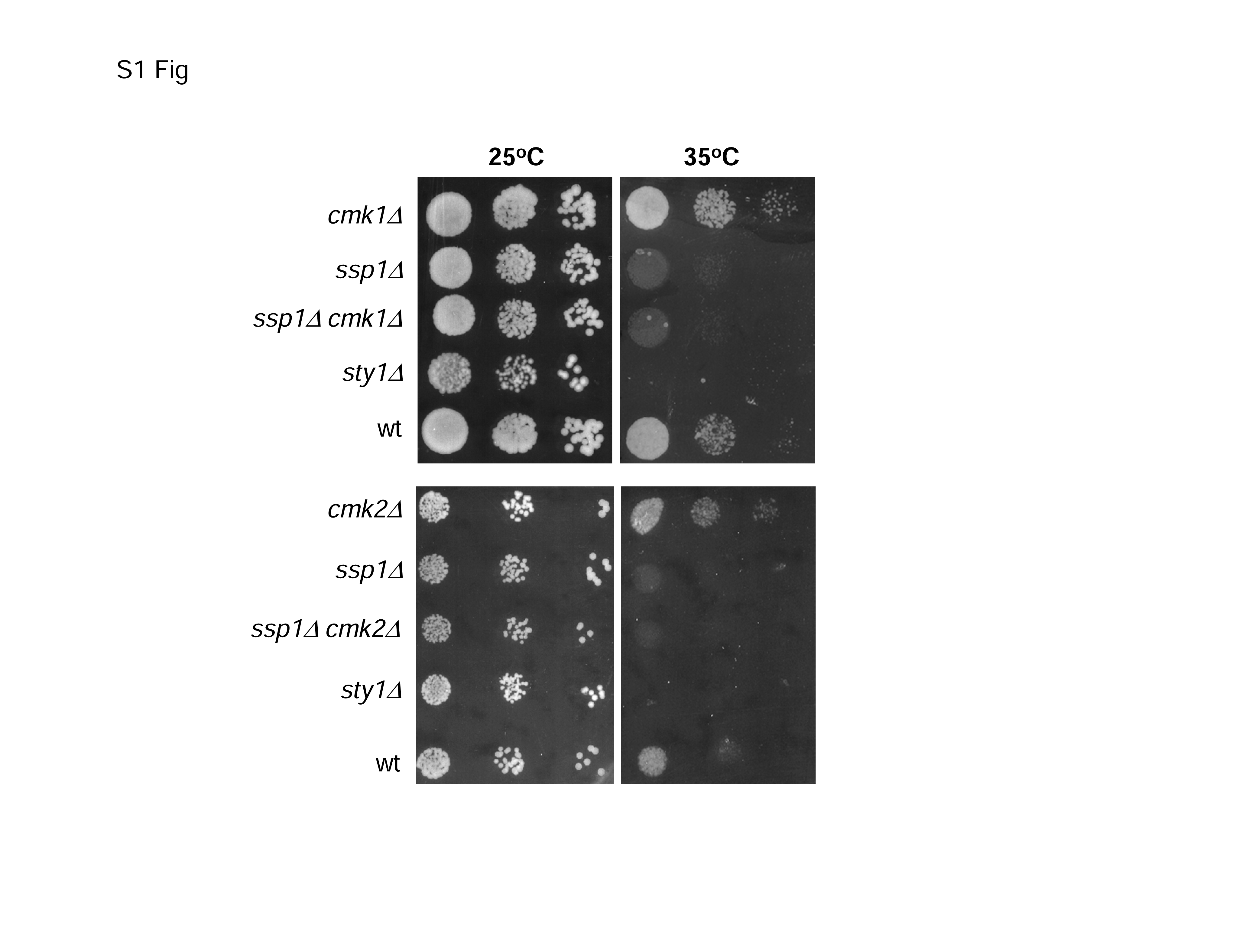

Supplement: S1 Fig — Wild type (wt), cmk1Δ. cmk2Δ, ssp1Δ, cmk1Δ ssp1Δ, cmk2Δ ssp1Δ and sty1Δ cells were grown in YES medium and spotted on YES plates and incubated for 3 days at 25°C and 35°C. (TIF) [file pone.0143037.s001.tif]

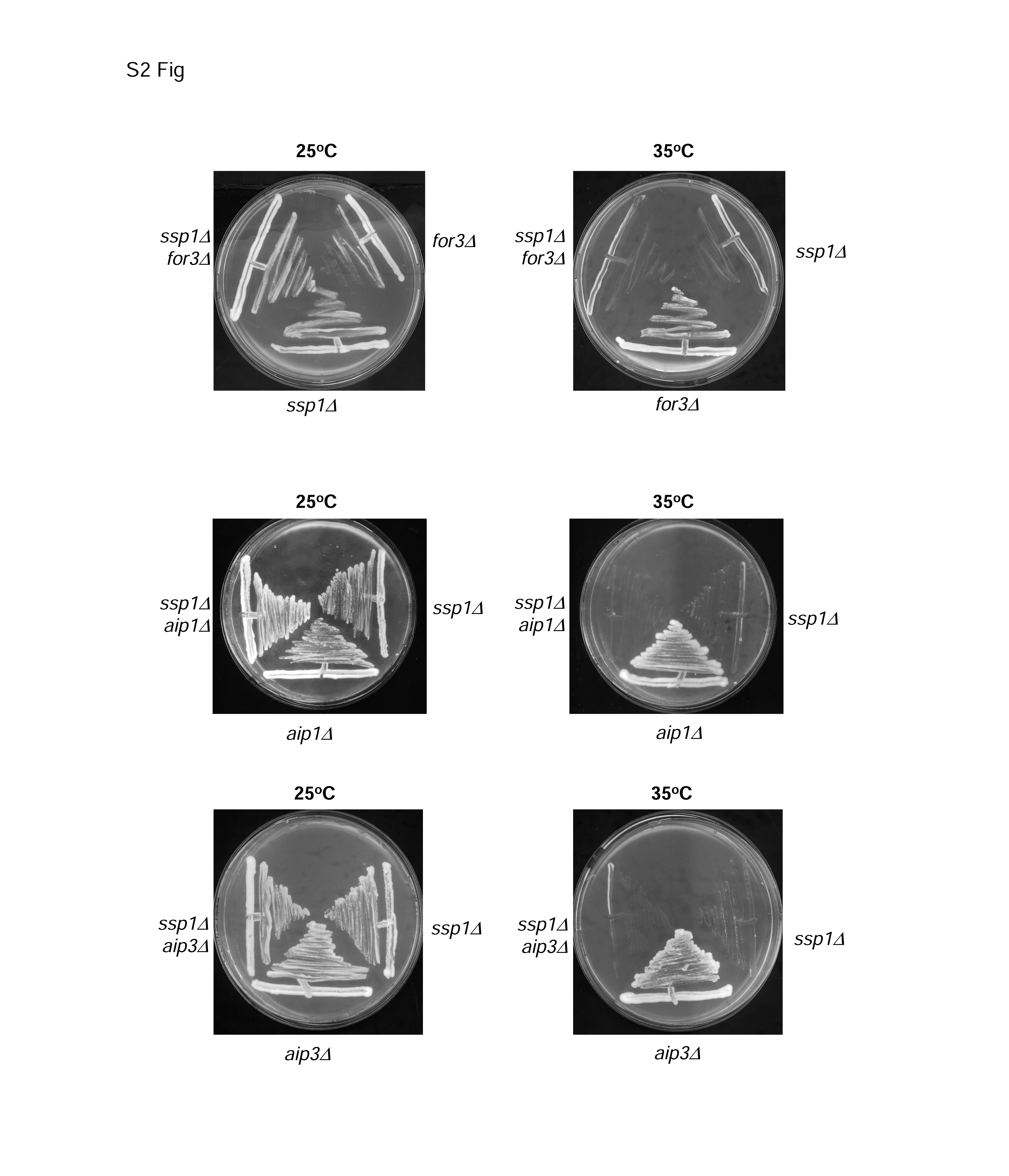

Supplement: S2 Fig — (A) ssp1Δ, for3Δ and ssp1Δ for3Δ cells were grown on YES plates for 3 days at 25°C and 35°C. (B) ssp1Δ, aip1Δ and ssp1Δ aip1Δ cells were grown on YES plates for 3 days at 25°C and 35°C. (C) ssp1Δ, aip3Δ and ssp1Δ aip3Δ cells were grown on YES plates for 3 days at 25°C and 35°C. (TIF) [file pone.0143037.s002.tif]
